# Whole-genome sequencing, phenotypic characterization, and antifungal susceptibility profiles of three *Aspergillus hortae* clinical isolates from Colombia

**DOI:** 10.1371/journal.pone.0342479

**Published:** 2026-02-17

**Authors:** Santiago Marin-Carvajal, Mariana Quiceno Torres, Maria Camila Zuleta, Susana Torres, Álvaro Rúa-Giraldo, Ana Maria García, Clayton L. Borges, Angela Maria López, Juan G. McEwen, Orville Hernández, Oscar Mauricio Gómez

**Affiliations:** 1 Cellular and Molecular Biology Unit, Corporación para Investigaciones Biológicas (CIB), Medellín, Colombia; 2 School of Microbiology, Universidad de Antioquia (UdeA), Medellín, Colombia; 3 School of Pharmaceutical Chemistry, Universidad de Antioquia, Medellín, Colombia; 4 Laboratory of Molecular Biology, Federal University of Goiás, Goiás, Brazil; 5 School of Medicine, Universidad de Antioquia, Medellín, Colombia; 6 MICROBA Research Group, School of Microbiology, Universidad de Antioquia, Medellín, Colombia; Leibniz-Institut fur Naturstoff-Forschung und Infektionsbiologie eV Hans-Knoll-Institut, GERMANY

## Abstract

The *Aspergillus* genus comprises fungi that are widely distributed in nature. Several *Aspergillus* sections are important for recycling organic matter; while the *Fumigati* section is classically associated with compost, the *Terrei* section also plays a relevant role in this process. *Aspergillus hortae* is a species from this section that has been isolated from clinical samples, but its role as a pathogenic agent is unclear. In this study, we analysed three clinical isolates of *A. hortae* from Colombia, initially misidentified as *A. terreus*. Morphological characterization at 26 °C and 37 °C confirmed its thermotolerant nature. Whole-genome sequencing enabled accurate species identification and revealed phylogenetic divergence from the reference genome. Antifungal susceptibility testing (EUCAST broth microdilution) showed intrinsic resistance to amphotericin B (MIC 2–4 mg/L) and susceptibility to azoles (MIC 0.25–1 mg/L). A mutation (M769K) in the MelA ortholog of a hypopigmented isolate, which had the lowest MIC value for AmB (2 mg/L), was identified. This study presents the morphological characterization, molecular typing through whole-genome analysis and identification of susceptibility profiles to azoles and amphotericin B of three clinical isolates of *A. hortae* from Colombia.

## Introduction

*Aspergillus* section *Terrei* represents an important infragenic taxonomic rank within the *Aspergillus* genus, grouping species that share close phylogenetic relationships and similar morphological traits. This section is noteworthy due to its biological diversity and ecological functions. Species belonging to section Terrei are mainly saprophytic, frequently occurring in soil and organic matter [1]. Furthermore, they are utilized in the industry as producers of secondary metabolites, including drugs and other bioactive compounds [2,3]. However, some isolates from this section are often isolated in clinical samples and linked with diseases in both human and animals [4]. One of the species described within the section *Terrei* is *Aspergillus hortae*, which is phylogenetically close to *A. terreus* [5]. *A. hortae* has been briefly mentioned in the literature as a human pathogenic species [4,6] and has been isolated from clinical samples such as ear secretions [5]. However, in Colombia *A. terreus* was the only species within the section *Terrei* reported in clinical samples. Currently, knowledge about the diversity of this fungus remains limited. To date, no published studies have provided a detailed genomic analysis of *A. hortae*, although a genome assembly is available in public databases (Mycocosm ID: 1307). Clinical case reports and epidemiological data are also limited to only a few isolates, underscoring its potential as an underrecognized pathogen [5]. As an organism with pathogenic capacity, it is necessary to acquire additional knowledge regarding its phenotypic and genotypic characteristics, as well as its geographical distribution.

At present, whole-genome sequence approaches have expanded the knowledge about the genetic characteristics of pathogens [7]. The application of next-generation sequencing technologies in *Aspergillus* is enabling a rapid, precise, and in-depth identification of the multiple species within this genus. These approaches enable simultaneous species identification, prediction of drug resistance, and discovery of genetic variants [8,9]. Despite the enormous risk that antifungal-resistant isolates from the genus *Aspergillus* can pose to human health, whole-genome sequences of this genus are still limited, particularly in non-*fumigatus Aspergillus* species [8].

The wide genotypic diversity present within the *Aspergillus* genus makes the section/species that the patient is exposed to a key parameter when selecting an appropriate antifungal treatment. This is because *Aspergillus* spp. do not have homogeneous susceptibility profiles [10]. In the section *Terrei*, for example, an intrinsic resistance to amphotericin B has been reported [4]. The issue of resistance to antifungal treatment is on the rise, with azoles in particular posing a worldwide challenge [11,12]. It is urgent to characterise the pathogenic species of the genus *Aspergillus* and understand their susceptibility profiles to different antifungal drugs.

Considering the reasons stated above, the aim of this study was to provide a morphological characterization, perform molecular typing through whole-genome analysis, and identify susceptibility profiles to azoles and amphotericin B of three clinical isolates of *A. hortae* from Colombia.

## Materials and methods

### Fungal isolates

Three clinical isolates of *Aspergillus* section *Terrei* were obtained from the Fungi Collection at the Corporación para Investigaciones Biológicas (CIB, Medellin; Colombia). The isolates were identified at the section level by microscopic identification technique based on morphological features [1]. These isolates were initially identified and reported as *A. terreus,* and were named as MC7, MC8, and MC10. Ethics committee approval was obtained from the local Ethics committee of the Corporación para Investigaciones Biológicas.

### Phenotypic characterization

After obtaining a pure culture, the isolates were inoculated at three points using a micropipette and an inoculum size of 1 μl per spot into petri dishes containing Malt Extract Agar (MEA; Scharlau), Potato Dextrose Agar (PDA; Millipore), Sabouraud Agar (SB; DIFCO), and Czapek Yeast Agar (CYA; Millipore) were incubated at 26°C and 37°C. All cultures, regardless of medium or temperature, were performed in three independent replicates and incubated for 7 days in the dark. After the incubation period, the macroscopic characters of the colonies and the microscopic characteristics on MEA were evaluated. Macroscopic characteristics including colony diameters and texture, obverse and reverse colony colours and exudates were determined. After 10 days of incubation, microscopic characters such as the shape of conidial heads, the presence or absence of metulae between vesicle and phialides, colour of stipes, and the dimension, shape, and texture of stipes, vesicles, metulae, phialides, and conidia were evaluated. Each isolate was morphologically characterised using standardised and recommended methods for laboratories working with *Aspergillus* spp*.*, as described by Samson et al. (2014) [1]. Photographic records of the colonies were obtained using a digital camera, and images of the microscopic structures were captured using the Leica ICC50 W microscope. The colony diameters were analysed in SPSS 24 software through a Klustal-Wallis test.

### Antifungal susceptibility test

The antifungal susceptibility test was carried out via a 2X microdilution broth method following EUCAST instructions (E.DEF 9.4) [13]. The antifungals evaluated in this study were, Voriconazole (VRC), Itraconazole (ITC), Posaconazole (POS) and Amphotericin B (AmB). To test antifungal susceptibility, a suspension of each isolate was prepared in RPMI 1640 broth medium. The four antifungals mentioned above were diluted in dimethyl sulfoxide (DMSO). The strains were tested against 10 concentrations (0.03–8 mg/L) in 96-well plates by 2x dilution and incubated at 35°C for 48 h. All experiments were performed in triplicate.

### DNA extraction

Isolates were cultured in Brain Heart Infusion (BHI) at 30°C, at 120 rpm. The biomass was collected during the exponential growth phase after 96 hours of incubation. Cell lysis was performed by mechanical disruption using liquid nitrogen. Genomic DNA was obtained using phenol/chloroform extraction, and RNA was eliminated by treatment with 10 μg of RNase A (Thermo Fisher Scientific, USA) for 120 min at 37°C [14]. DNA quality was evaluated using a NanoDrop 2000 spectrophotometer (Thermo Fisher Scientific, USA) to determine concentration and purity, using the default setting (1 OD = 50 mg/mL dsDNA). The 260/280 absorbance ratio was used as a quality parameter to ensure DNA purity. In addition, the integrity of the DNA recovered from isolates was evaluated through 1% agarose gel electrophoresis.

### Whole genome sequencing

Library preparation was carried out using Illumina Nextera DNA Library Preparation Kit (Illumina Inc. San Diego, CA, USA), with 500 ng of DNA per sample. Sequencing was performed using the second generation sequencing technology Illumina Xten (Inc. BGI Hong Kong), generating 150-bp paired-end sequencing. The samples were run on one sequencing line on the Illumina platform, generating around 7 million paired-end reads per isolate and producing an average genome coverage of 30X. All relevant data are available within the manuscript and its Supporting Information files. Raw sequencing data have been deposited in the NCBI Sequence Read Archive (SRA) under BioProject accession number PRJNA975750. The corresponding BioSample accession codes are SAMN35344990, SAMN35344991, and SAMN35344992. These data are publicly accessible without restriction.

### Pre-assembly analysis

FastQC version 0.11.8 program was used to analyse the FASTQ quality code of the short paired-end raw reads [15], considering Phred scores above 30 as the quality threshold to ensure high sequence accuracy. Trimmomatic version 0.39 was employed to filter out low-quality (Q < 30) sequences and adapters [16].

### Genome assembly

The SPAdes software version 3.10 [17] with the BayesHammer module for error correction [18] was used to process data. *De novo* assembly of the short reads (2x150) was performed, and iterative k-mer lengths (21, 33, 55, 77 bp) were used to take advantage of the paired-end reads. The draft genome assembly’s quality was evaluated using QUAST version 5.2.0 [19]. Three parameters were utilized to verify the quality of each genome: the average coverage of paired-end reads, histograms of the distribution of the percentage of Guanine-Cytosine (% GC), and sequence alignments using the genes of the *A. hortae* IBT 26384 as a reference. The genomes available were downloaded from the JGI MycoCosm database (https://genome.jgi.doe.gov/programs/fungi/index.jsf).

### Gene homology analysis

The *Aspergillus terreus* model was used for *ab initio* gene prediction using Augustus version 3.0.1 [20]. The predicted protein sets were then compared using OrthoFinder version 2.0.9 pipeline [21] to analyse sequence homology with the representative genomes of *Aspergillus* section *Terrei* reported in the databases (S1 Table).

### Species identification by barcoding

To identify the species of *Aspergillus* isolates, ITS, *CaM*, and *BenA* sequences were identified in the assembly using a local blastn search. The query reference sequences from GenBank®, *A. terreus BenA* (EF669520.1), *A. hortae CaM* (KP987054.1), and *A. hortae* ITS (OL711861.1), were used. A web blastn search was then performed on these sequences in the Nucleotide Collection (nr/nt) of NCBI and EMBL-bank databases with default settings. The workflow used for species identification by barcoding is shown in Supplementary S1 Fig.

### Phylogenetic analysis

The resulting sequences were aligned with reference sequences from *Aspergillus* section *Terrei* (S2 Table) using the ClustalW version 2.1 program. For phylogenetic reconstruction, the Maximum Likelihood (ML) method [22] was employed with IQtree version 1.4.4 software, and the best nucleotide substitution model was estimated. Phylogenetic analysis was conducted using individual genes and a concatenated matrix with *BenA* and *CaM* markers. The Bootstrap method with 1000 replications was used to evaluate the internal branches.

### Whole-genome single-nucleotide polymorphism (snp) calling and phylogenetic analysis of *A. terreus* clade

We download available Illumina reads of species from this clade (SRA NCBI database) (S3 Table). Each of the 10 Illumina data sets was independently aligned to the *A. terreus* reference genome using BWA version 0.5.9 [23] with default settings. SNPs and indels were called with Pilon version 1.4 using the haploid ploidy default setting. Variant call format (VCF) files were filtered using VCFtools version 0.1.1 (minimum depth 4). Alignments were constructed from SNP matrices extracted from the VCF files.

## Results

### Morphological analysis

**Fig 1:**
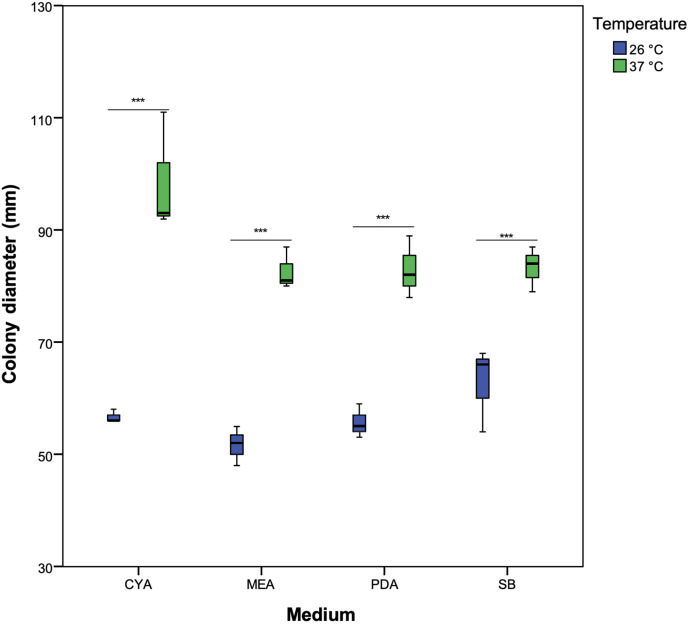
Radial growth box plot of *A. hortae* clinical isolates in different culture media and temperatures. (t-test) *** statistical significance p < 0.001.

After incubating the colonies at both 26°C and 37°C for seven days in darkness, it was observed higher biomass production in all cultures at 37°C. The cultures grown at 26°C had a lower colony diameter (56.67 ± 5.60 mm), with significantly better growth (p < 0.05) in all evaluated media at 37°C (86.92 ± 9.07 mm). Additionally, the highest colony diameter was observed in CYA at 37°C (98.7 ± 10.69 mm) while the lowest was observed in MEA at 26°C (51.67 ± 3.51 mm) (Fig 1). As the colony matures, a dark center with a lighter periphery becomes evident. The isolated MCA7 strain shows lighter colony pigmentation ranging from white to light beige. No exudate formation was observed in any of the strains. On the reverse side of the colony, the formation of pigments ranging from light brown to dark brown is observed. These morphological characteristics are consistent with what has been described in the literature for *Aspergillus* section *Terrei* [5].

**Fig 2:**
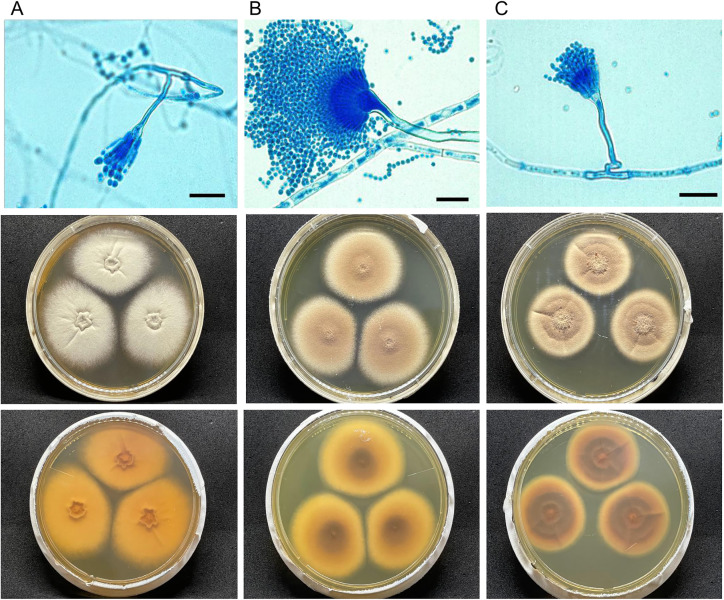
Morphological characteristic of clinical isolates of *A. hortae.* MCA-7 **(A)**, MCA-8 (**B**) and MCA-10 **(C)**. Microscopic view Lactophenol cotton blue mount in 100X (top) and Macroscopic view of colonies in MEA (down). Scale bars = 10 μm. Reproduced from Marin-Carvajal et al., 2024, (https://doi.org/10.20944/preprints202409.0031.v1) with permission from Santiago Marín-Carvajal. Original copyright 2024.

The colonies grown on CYA and MEA culture media at 26°C exhibit compact, columnar-shaped heads. The conidiophores are short, hyaline, and have smooth walls. *A. hortae* vesicles take the form of half-heads with extensive cylindrical metulae, from which the phialides emerge. The conidia are round, hyaline, and have smooth walls. When grown on CYA and MEA culture media at 37°C, the colonies display a dense cottony appearance with a beige to cinnamon brown colour. (Fig 2).

### Genome assembly and analysis

To compare genome structures and gene contents between Aspergilli, we sequenced, assembled, and predicted the proteins of the three *A. hortae* isolates included in this study. In the assemblies, we found characteristics like those reported for the *A. hortae* reference genome (Mycocosm ID: 1307), including a genome size ranging from 29.86–31.90 Mb and a GC content of 52.05–52.19% (S3 Table). The *ab initio* predictions showed a range of 13,120–13,300 predicted protein-coding genes, with a high proportion (>99%) of homologs found in the genus *Aspergillus*. In *Aspergillus* section *Terrei,* 4,677 single-copy orthologues were identified. The phylogenetic tree of *Aspergillus* section *Terrei* using aminoacid sequences is shown in supplementary S2 Fig.

Using the local BLASTn tool, the sequences of the markers (ITS, *CaM, BenA*) were found within the assemblies, which are commonly used for phylogenetic analysis and identification of the *Aspergillus* genus [1]. Only one copy of these genes was found in each of the assemblies. Subsequently, a web search was conducted to compare these sequences with the databases, leading to the successful identification of the species as *A. hortae.* The best hit for Aspergillus hortae was with the BenA gene (100% identity and 99.81% coverage), while for the ITS and CaM genes, similar hits were observed with the species *Aspergillus terreus*. This result showed that the isolates had a high probability of being classified as *A. hortae*.

**Fig 3:**
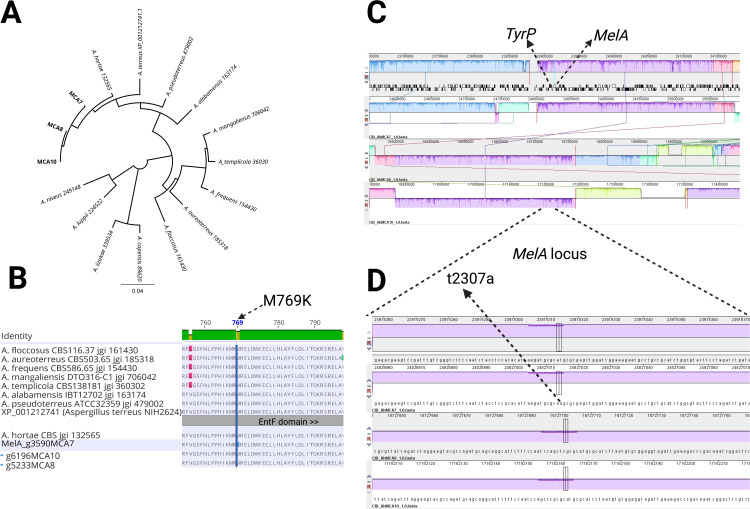
Genomic variation analysis in *A. hortae.* **(A)** Neighbor joining phylogenetic tree of MelA in *Terrei* and *Flavipedes* sections. **(B)** Alignment of 12 MelA (XP_001212741) predicted orthologs from the *Aspergillus* section *Terrei,* highlighting a K679M substitution in the hypopigmented isolate MCA7. **(C)** Alignment of whole genome sequences of *A. hortae*. Three clinical isolates were aligned to the genome reference Asphor1_AssemblyScaffolds, and Mauve analysis reveals possible inverted regions (red R) in two of the clinical isolates. These regions contain the melanin associated genes (*MelA* and *TyrP* orthologs from *A. terreus*). **(D)** *MelA* locus exhibits a SNP, with an A to T substitution, in the MCA7 isolate.

The alignment of three *de novo* assemblies from Colombian isolates to the reference genome (Asphor1_AssemblyScaffolds) showed conserved synteny when comparing the Locally Collinear Blocks (LCB) generated in mauve. However, in the alignment, LBC in the reverse panel (R) is observed in the three new assemblies, and these are relatively conserved in them, mainly in MCA8 and MCA10 isolates (Fig 3). After homology analysis using Orthofinder, we identified the orthologs associated with melanin production in *A. terreus*, MelA (XP_001212741), and TyrP (XP_001212742.1). These two proteins are conserved in the *Terrei* and *Flavipedes* sections, and TyrP orthologs were found in almost all evaluated species, while MelA orthologs were found only in 13 species (Fig 3A). MelA possesses a conserved Thioesterase domain of type I polyketide synthase (EntF), however, during protein alignment, a K769M point mutation was observed in the hypopigmented isolate MCA7. The Methionine at position 769 is conserved in all species, while MCA7 is the only isolate with a change in this amino acid (Fig 3B).

When the whole genome is aligned to *A. hortae*, *TyrP* and *MelA* are located contiguously within a LCB in the alignment of the four *A. hortae* genome assemblies. However, the two highly pigmented isolates (MCA8 and MCA10) showed the LBC with melanin-associated genes in an inverted orientation (Fig 3C). Finally, we zoomed into the *MelA* locus*,* where the nucleotide substitution t2307a (XM_001212741.1) was found in the MCA7 isolate (Fig 3D).

### Phylogenetic analysis

**Fig 4:**
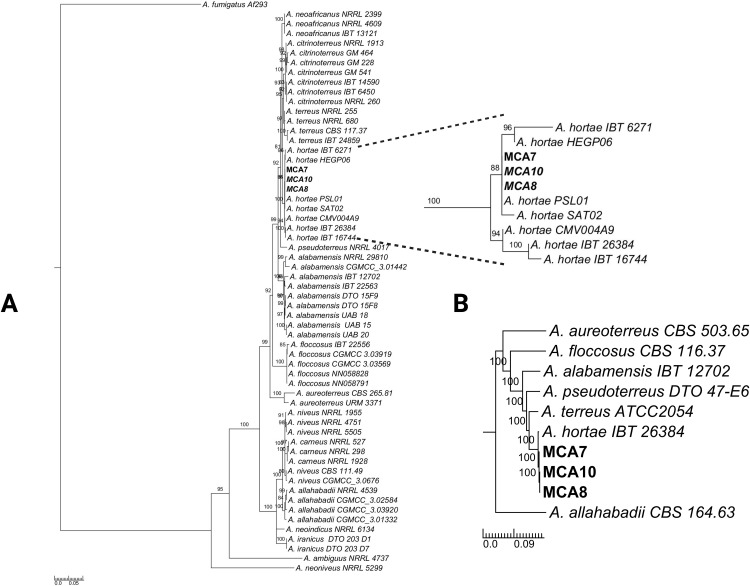
Phylogenetic Analysis. Phylogenetic tree of *Aspergillus* section *Terrei* using concatenated sequences from the *BenA-CaM* markers, *A. fumigatus* Af293 *CaM* was utilized as an outgroup. **(A)**. Phylogenomic tree of *A. terreus* clade based on 124,027 SNPs position **(B)**. The trees were inferred using the Maximum Likelihood method. The numbers close to branches are % of supported bootstrap after 1,000 replications. Colombian clinical isolates of *A. hortae* are shown in bold.

Our best phylogenetic reconstruction was achieved using concatenated sequences of *BenA* and *CaM* in a partitioned matrix of 59 OTUs and 1344 characters. Modelfinder was used to determine the best nucleotide substitution model for each partition (S4 Table). The three isolates were grouped with reference isolates from *A. hortae*, creating a monophyletic clade (Bs = 88%) with IBT 6271, IBT 6271, HEGP06, PSL01, and SAT02. The reference genome strain IBT 26384 is observed as an outgroup in another monophyletic clade (Bs = 94%) with strains IBT 16744 and CMV004A9. The *A. hortae* species is closely related to the clade formed by *A. terreus* and *A. citrinoterreus* species (Fig 4). The phylogenetic reconstruction using sequences from ITS marker, does not provide clear genotypic differentiation between *A. hortae* and other species from *Aspergillus* section *Terrei* (S3 Fig). In the phylogenetic tree of the clade *A. terreus*, the three Colombian isolates of *A. hortae* cluster with the reference strain IBT 26384 and are positioned as a sister species to *A. terreus*, with *A. pseudoterreus* serving as the outgroup.

### Antifungal susceptibility testing

The MIC values with VRC were varied, with two isolates (MCA7 and MCA8) demonstrating a result of 0.5 mg/L and MCA10 having twice this value (1 mg/L). In the case of ITC, the MIC were consistent at 1 mg/L across all three isolates. Similarly, the MIC of POS were consistent across all three isolates, with the lowest MIC values (0.25 mg/L). In contrast, the three *A. hortae* isolates exhibited high MIC values upon AmB exposure (2–4 mg/L), with MCA7 displaying the lowest MIC value (2 mg/L), whereas the other isolates showed twice the MIC value (4 mg/L). These results are based on three independent replicates, in which no variation among the values was observed (Table 1).

**Table 1:** Antifungal susceptibility test of *Aspergillus hortae* isolates.

		VRC		ITC		POS		AmB	
Species	Isolate	MIC	INT	MIC	INT	MIC	INT	MIC	INT
***A. hortae***	**MCA-7**	0.5	S	1	S	0.25	S	2	R
***A. hortae***	**MCA-8**	0.5	S	1	S	0.25	S	4	R
***A. hortae***	**MCA-10**	1	S	1	S	0.25	S	4	R

VRC: Voriconazole. ITC: Itraconazole, POS: Posaconazole, AmB: Amphotericin B. S: Sensible. R: Resistant. MIC: Minimum Inhibitory Concentration (mg/L). INT: interpretation.

## Discussion

In this study, we described the first report of susceptibility profiles of *A. hortae* clinical isolated in Colombia. A key limitation of this study concerns the origin of the analyzed samples. The clinical isolates were obtained from an archival strain repository within a fungal collection, for which patient information and infection context were incomplete. As a result, it was not possible to establish a direct association between the phenotypic and genotypic traits of the isolates and the corresponding clinical manifestations. This limitation is recognized to provide a balanced interpretation of the results and to prevent overstating their clinical significance.

Upon analysing the MIC of the isolates MCA-7, MCA-8 and MCA-10, and comparing them with the cut-off established in EUCAST, we confirmed that these isolates exhibit intrinsic resistance to AmB as previously reported [24]. Isolates from different species of *Aspergillus* section *Terrei*, including *A. hortae*, display a high tolerance to this antifungal *in vitro* [4,24]. However, the AmB susceptibility profiles of the isolates were not uniform; MCA7 exhibited higher susceptibility to AmB than the other two isolates. Imbert *et al*, in 2018 also reported two *A. hortae* French clinical isolates with low AmB MIC values (0.25 mg/L) [6], indicating genetic variability within this species. The AmB resistant phenotype has been linked to changes in the cell membrane of the fungus alterations in the expression of membrane transporters, or modifications in the structure of the drug target [25]. Nevertheless, the exact resistance mechanisms in these species are still unclear. Further investigation of the genomes of a high number of species from this section, as well as the phylogenetically related sections *Fumigati* and *Flavi*, whose susceptibility profiles were heterogeneous, could aid in the elucidation of this matter [26,27].

The fact that we found isolates with heterogeneous AmB resistance and pigmentation levels indicates genetic diversity in this fungal species. Interestingly, the isolate with the lowest pigmentation had the lowest MIC value with AmB and harboured a mutation in a melanin pathway gene. In *Cryptococcus* spp., high melanin pigmentation isolates are associated with decreasing AmB susceptibility [28]. Similarly, in *W. dermatitidis*, strains with *PSK1* gene knocked out displayed increased susceptibility to both AmB and VRC [29]. This suggests that AmB susceptibility in *A. hortae* could also be associated with melanin production. Geib et al, 2016 demonstrated in *A. terreus* that the pigment produced by MelA and TyrP protects conidia from biotic and abiotic stress factors [30]. However, the role of melanin in AmB resistance was not evaluated. Future studies could investigate the susceptibility of *MelA* and *TyrP* knockout strains from section *Terrei* to AmB. This study could help to understand the importance of this pigment in the antifungal resistance.

The three isolates were susceptible to the three evaluated azole drugs (ITC, POS, VRC). ITC and POS showed the lowest MIC values, in agreement with previous studies on this fungal species [11,24]. Meanwhile, VRC displayed a heterogeneous profile. This patron could be considered as a caution sign. Despite the fact that all isolates evaluated are classified as susceptible to this drug, its VRC resistance has been observed in other species that exhibit the same profile [11]. The isolated (MCA10) with VRC higher MIC value also had differences in the locally collinear blocks (LCB) profiles with respect to the other isolates (MCA7 and MCA8), when their draft assemblies were aligned to the reference genome. Intra-chromosomal rearrangements are a molecular mechanism recently associated with antifungal resistance evolution in *Candida auris* [31].

Here, we reported three new genome assemblies from clinical isolates of *A. hortae,* which exhibit significant phylogenetic divergence from the reference genome strain (IBT 26384, a clinical isolate from Brazil). It is noteworthy that this is the first time that genomic variability has been found in this *Aspergillus* species. Steenwyk et al. (2022), using WGS analysis, demonstrated an extensive misidentification of species and low novel lineages detection in the genus *Aspergillus* when only a few barcode markers were used [23]. Despite the phenotypic differences, the phylogenetic reconstruction revealed a high degree of genomic similarity among the three isolates. These phenotypic differences are more likely explained by underlying sequence variation across the genome, although potential chromosomal structural variation in *A. hortae* cannot be ruled out. Further analyses using long-read sequencing technologies, which provide greater precision and resolution for detecting structural variants, will be required to clarify these differences [32].

At the moment, the whole genome data for this species continues to be limited for phylogenomic reconstruction to examine the genetic diversity within this species. The species tree generated after homology analysis from section *Terrei* only showed that the three Colombian isolates are related to the only available *A. hortae* reference genome, whereas the *BenA-CaM* phylogeny with 10 isolates showed evidence of genetic divergence from this isolate. Further research with more isolates sequenced at genome level could be conducted to elucidate the true evolutive history and genetic diversity of *A. hortae*.

On the other hand, the ITS barcode marker has been used as a panfungal molecular test in the diagnostic laboratory [33,34]. However, it showed very low-resolution power in the *A. terreus/ A. hortae/ A. citrinoterreus* clade. It may have overestimated the epidemiology of *A. terreus* impeded our understanding of the actual impact on health of new cryptic species, such as *A. hortae*. The epidemiology of Aspergillosis has changed in recent years, with the emergence of new species, which could be attributed more to the improvement of typing techniques that have allowed for accurate classification [23,35]. Salem-Bango et al. (2023) propose the use of WGS in specialized laboratories to accurately identify *Aspergillus* species [9]. We agree that the adoption of WGS could be an effective solution for correct species identification in *Aspergillus* section *Terrei*, and we also suggest using at least the *BenA* as a second barcode marker for more precise identification of species within the section.

Unlike most Ascomycetes, which typically produce dihydroxynaphthalene (DHN) melanin as a defense mechanism against various conditions, *A. terreus* has been described to synthesize its melanin from 4-hydroxyphenylpyruvic acid, which is converted into aspulvinone E through the action of the MelA enzyme. This process leads to the formation of a distinct type of melanin known as Asp-melanin [36]. Geib et al. (2016) reported that deletion of the *melA* gene (ATEG_03563) in *A. terreus* is linked to the loss of conidial pigmentation [30]. In the present study, we describe for the first time a point mutation, K769M, in the *melA* gene of *A. hortae*. This amino acid substitution could potentially impair MelA enzymatic function, providing a plausible explanation for the hypopigmented phenotype observed in the MCA7 strain. Furthermore, MCA7 exhibited a lower minimum inhibitory concentration (MIC) to AmB B compared to the typically pigmented MCA8 and MCA10 strains, suggesting that the loss of melanin by this mutation may influence antifungal susceptibility. It is important to note that this is a descriptive finding, and the association between the K769M mutation and reduced AmB MIC remains speculative. Additional experiments using targeted mutants are required to confirm whether this mutation directly affects susceptibility to AmB.

Our findings on growth at 37 °C show concordance with previous reports describing *A. hortae* as a thermotolerant fungal species, a critical characteristic for microbial pathogens to thrive in human and animal hosts. This characteristic demonstrates how this species has evolved to adapt to the stress of growth in the host [37,38], and is also linked to virulence factors in other fungal pathogens, particularly in *A. fumigatus* [39], which tolerates up to 60°C, the upper temperature limit for eukaryotic organisms [40]. Lacker et al. (2019) also demonstrated that *A. hortae* at 37 °C exhibited the highest growth rates, and some isolates had high virulence potential in *Galleria mellonella* larvae [41]. These findings indicate that *A. hortae* could be an emergent human pathogenic fungal species. Furthermore, there is significant phenotypic and genetic variability among the isolates of this species, requiring further exploration.

## Conclusions

This study presents the first morphological, genomic, and antifungal susceptibility of clinical *Aspergillus hortae* isolates in Colombia. We acknowledge that this study is limited by the analysis of only three *A. hortae* isolates, which restricts the generalizability of our conclusions. Nevertheless, as *A. hortae* is an emerging pathogen with few cases reported in the literature, these findings provide valuable information and contribute to the understanding of this pathogen. The species exhibits thermotolerance, pigmentation variability, and heterogeneous antifungal responses, supporting its classification as an emerging pathogen. Genomic divergence revealed by WGS highlights the existence of potential regional lineages and the limitations of traditional identification methods. The observed association between pigmentation, MelA mutation, and AmB susceptibility opens new research avenues into resistance mechanisms in *A. hortae*; however, this is a descriptive finding, and further studies using targeted mutants are needed to confirm whether this mutation directly affects AmB susceptibility. Such studies aim to expand our understanding of pathogens and their reactions to current drug therapies, with the ultimate goal of enabling safer and more efficient dosage strategies for patient treatment.

## Supporting information

S1 Fig Bioinformatic workflow for species identification. (TIFF)

S2 Fig Phylogenetic tree of *Aspergillus* section *Terrei* using aminoacid sequences. (TIFF)

S3 Fig Phylogenetic tree of *Aspergillus* section *Terrei* using sequences from the ITS marker. (TIFF)

S1 Table Reference Proteomes used in OrthoFinder. (PDF)

S2 Table *Aspergillus* section *Terrei* SRA codes for phylogenomic reconstruction. (PDF)

S3 Table Assembly metrics of the sequences from strains MCA-7, MCA-8 y MCA-10. (PDF)

S4 Table List of best-fit models per partition for phylogenetic reconstruction. (PDF)
