# Supplementary figures and images for: Whole-genome sequencing, phenotypic characterization, and antifungal susceptibility profiles of three Aspergillus hortae clinical isolates from Colombia
Source: PLoS One. 2026 Feb 17;21(2):e0342479. doi: 10.1371/journal.pone.0342479 (PMC12912593; doi:10.1371/journal.pone.0342479)

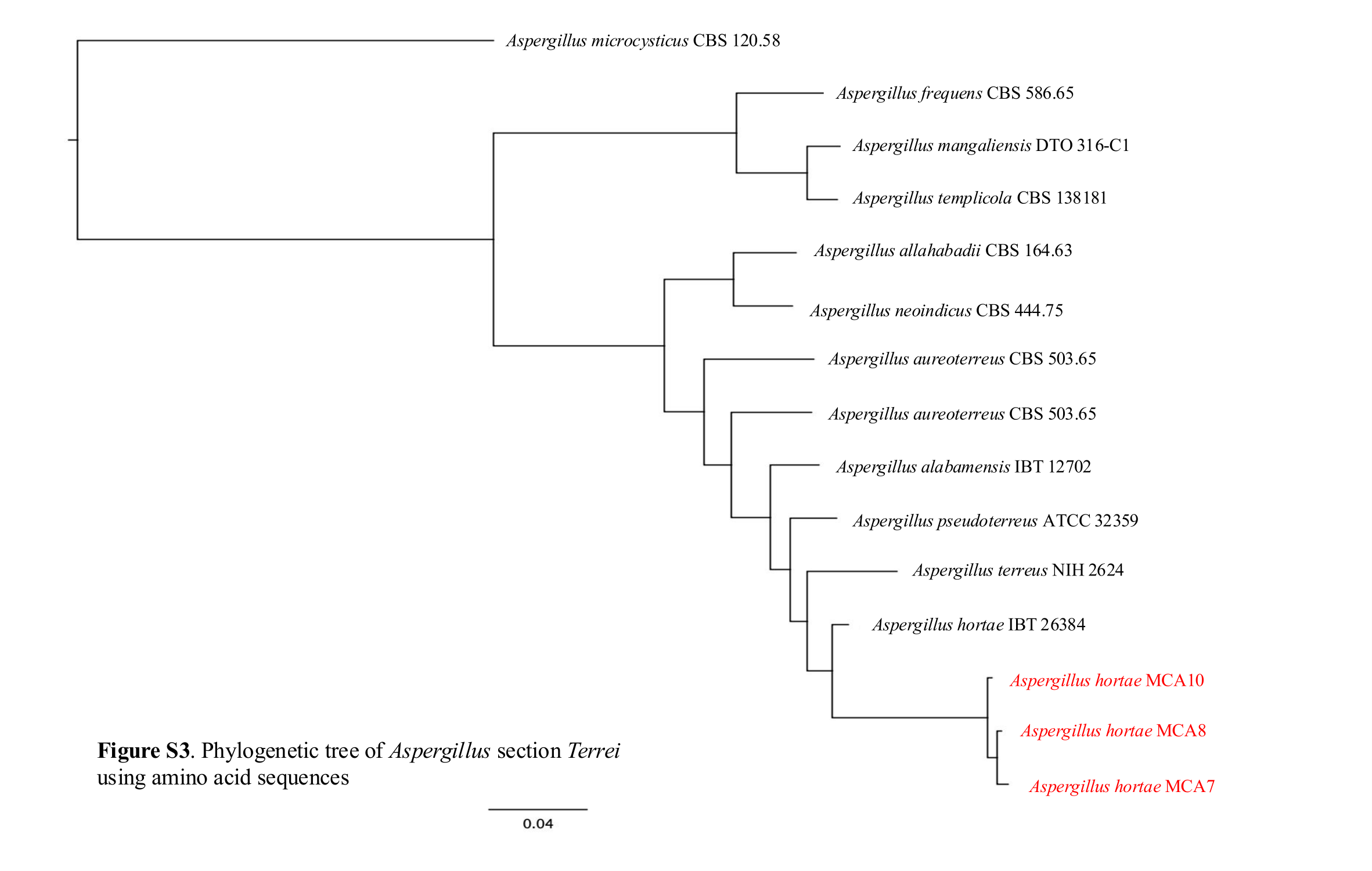

Supplement: S2 Fig — (TIFF) [file pone.0342479.s002.tiff]

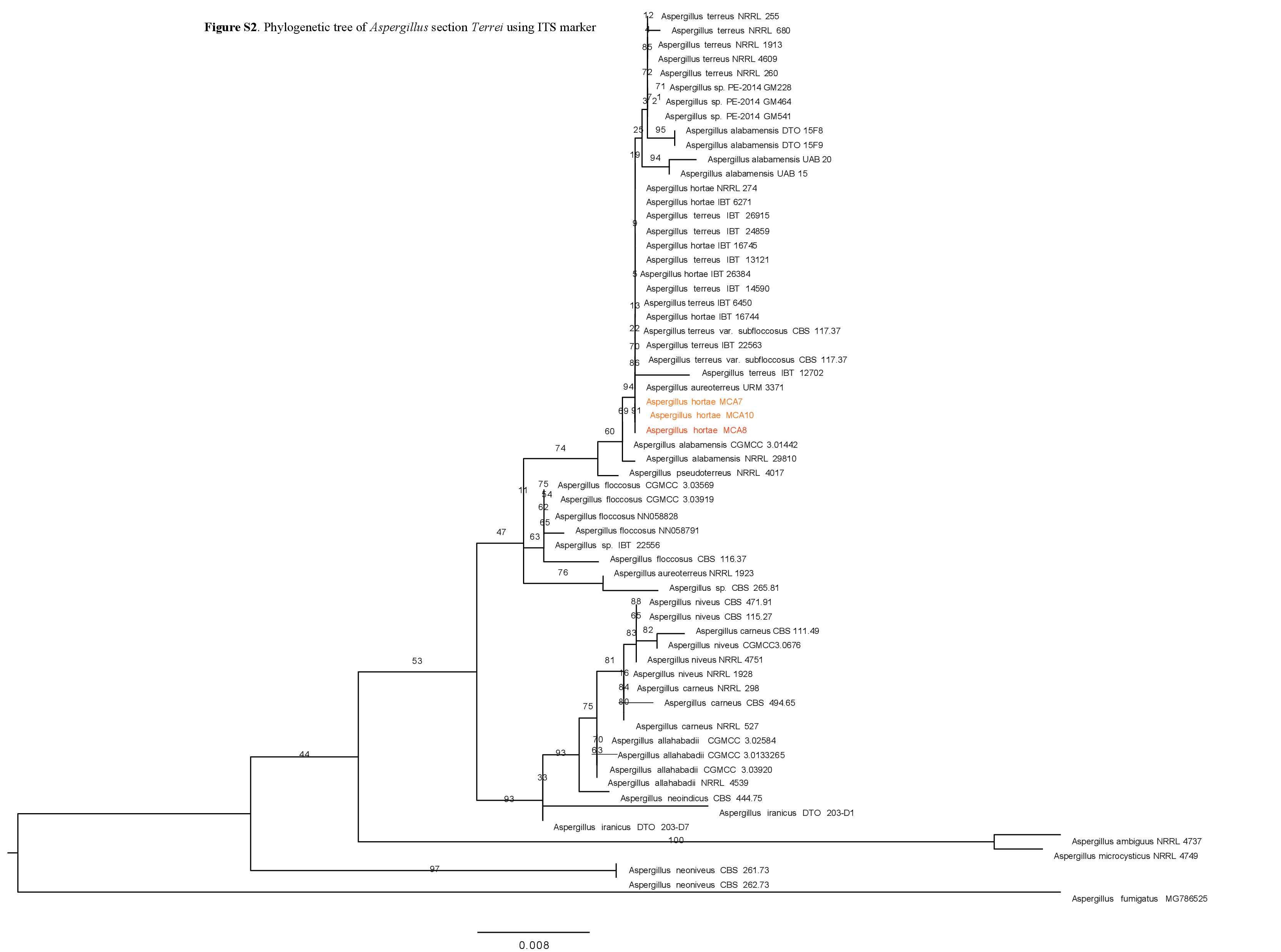

Supplement: S3 Fig — (TIFF) [file pone.0342479.s003.tiff]
