# Supplementary material for: Whole-genome sequencing, phenotypic characterization, and antifungal susceptibility profiles of three Aspergillus hortae clinical isolates from Colombia
Source: PLoS One. 2026 Feb 17;21(2):e0342479. doi: 10.1371/journal.pone.0342479 (PMC12912593; doi:10.1371/journal.pone.0342479)
Supplement: S1 Table — (PDF) [file pone.0342479.s004.pdf]

Table S1. Reference Proteomes used in OrthoFinder			
**Species**	**Strain/Isolate**	**Access number**	**Source**
A. terreus	NIH2624	GCF_000149615.1_ASM14961v1_protein.faa.gz	NCBI
A. pseudoterreus	ATCC 32359	Asppseute1_GeneCatalog_proteins_20210719.aa.fasta	Mycocosm*
A. mangaliensis	DTO 316-C1	Aspman1_GeneCatalog_proteins_20210405.aa.fasta	Mycocosm*
A. floccosus	CBS 116.37	Aspflo1_GeneCatalog_proteins_20170325.aa.fasta	Mycocosm*
A. alabamensis	IBT 12702	Aspala1_GeneCatalog_proteins_20160910.aa.fasta	Mycocosm*
A. allahabadii	CBS 164.63	Aspall1_GeneCatalog_proteins_20160910.aa.fasta	Mycocosm*
A. aureoterreus	CBS 503.65	Aspaur1_GeneCatalog_proteins_20160910.aa.fasta	Mycocosm*
A. hortae	IBT 26384	A_hortae_IBT26384.aa.fasta	Mycocosm*
A. frequens	CBS 586.65	Aspfre1_GeneCatalog_proteins_20170405.aa.fasta	Mycocosm*
A. mangaliensis	DTO 316-C1	Aspman1_GeneCatalog_proteins_20210405.aa.fasta	Mycocosm*
A. neoindicus	CBS 444.75	Aspneoi1_GeneCatalog_proteins_20170406.aa.fasta	Mycocosm*
A. templicola	CBS 138181	Asptem1_GeneCatalog_proteins_20191030.aa.fasta	Mycocosm*

* These sequence data were produced by the US Department of Energy Joint Genome Institute https://www.jgi.doe.gov/ in collaboration with the user community.
