# Supplementary material for: Whole-genome sequencing, phenotypic characterization, and antifungal susceptibility profiles of three Aspergillus hortae clinical isolates from Colombia
Source: PLoS One. 2026 Feb 17;21(2):e0342479. doi: 10.1371/journal.pone.0342479 (PMC12912593; doi:10.1371/journal.pone.0342479)
Supplement: S2 Table — (PDF) [file pone.0342479.s005.pdf]

Table S2 *Aspergillus* section *Terrei* SRA code for phylogenomic reconstruction

Aspergillus allahabadii CBS 164.63	SRR8397341
Aspergillus floccosus CBS 116.37	SRR10507650
Aspergillus pseudoterreus DTO 47-E6	SRR10511973
Aspergillus alabamensis IBT 12702	SRR8399219
Aspergillus aureoterreus CBS 503.65	SRR8399223
Aspergillus frequens CBS 586.65	SRR10513659
Aspergillus templicola CBS 138181	SRR12142453
Aspergillus terreus strain ATCC2054	SRR9691935
