# Supplementary material for: Whole-genome sequencing, phenotypic characterization, and antifungal susceptibility profiles of three Aspergillus hortae clinical isolates from Colombia
Source: PLoS One. 2026 Feb 17;21(2):e0342479. doi: 10.1371/journal.pone.0342479 (PMC12912593; doi:10.1371/journal.pone.0342479)
Supplement: S3 Table — (PDF) [file pone.0342479.s006.pdf]

**Table S3.** Assembly metrics of the sequences from strains MCA-7, MCA-8 y MCA-10

| | **MCA-7** | **MCA-8** | **MCA-10** |
|---|---|---|---|
| Genome accession | **JASVVZ000000000** | **JASVVY000000000** | **JASVVX000000000** |
| **Size (Mb)** | 29,86 | 31,17 | 31,90 |
| **Coverage** | 31X | 29X | 29X |
| **GC (%)** | 52.19 | 51.98 | 52.05 |
| **Number of contigs** | 148 | 213 | 362 |
| **Largest Contig (Mb)** | 2,25 | 2,6 | 2,8 |
| **N50 (Mb)** | 1,2 | 1,3 | 1,04 |
| **L50 (contig)** | 10 | 10 | 11 |
| **N75 (Mb)** | 0,647 | 0,55 | 0,513 |
| **L75 (contig)** | 18 | 17 | 21 |
