# Supplementary material for: Whole-genome sequencing, phenotypic characterization, and antifungal susceptibility profiles of three Aspergillus hortae clinical isolates from Colombia
Source: PLoS One. 2026 Feb 17;21(2):e0342479. doi: 10.1371/journal.pone.0342479 (PMC12912593; doi:10.1371/journal.pone.0342479)
Supplement: S4 Table — (PDF) [file pone.0342479.s007.pdf]

Table S4. List of best-fit models per partition for phylogenetic reconstruction.

ID number	Gene/Marker	Partition Name	Evolution Model
1	BenA	BenA_Intron1	K2P+G4
2	BenA	BenA_Exon2	K2P+I
3	BenA	BenA_Intron2	K2P+I
4	BenA	BenA_Exon3	K2P+I
5	BenA	BenA_Intron3	K2P+I
6	BenA	BenA_Exon4	TIM3e+G4
7	CaM	CaM_Exon1	K2P
8	CaM	CaM_Intron1	K2P+G4
9	CaM	CaM_Exon2	TNe
10	CaM	CaM_Intron2	K2P+G4
11	CaM	CaM_Exon3	TNe+I
12	CaM	CaM_Intron3	K2P+G4
13	CaM	CaM_Exon4	TIM2e+G4
14	CaM	CaM_Intron4	K2P+I
15	CaM	CaM_Exon5	K2P
